# Bilateral synchronous carcinoma breast- a rare case presentation

**DOI:** 10.1186/s40064-015-0953-3

**Published:** 2015-04-22

**Authors:** Vijay Kumar Shankarrao Kappikeri, Akshay Mahesh Kriplani

**Affiliations:** Department of Surgery, M.R. Medical College, Sedam Road, Gulbarga, Karnataka 585101 India

**Keywords:** Bilateral breast carcinoma, Synchronous breast carcinoma, Lobular carcinoma, Infiltrating ductal carcinoma, Toilet mastectomy, Simple mastectomy

## Abstract

**Introduction:**

Bilateral Breast Carcinoma (BBC) is a rare entity with incidence of synchronous carcinoma being 2-5% of all breast malignancies, which is much less than metachronous carcinoma. Synchronicity/ metachronicity are usually associated with local and lymphatic spread and with blood–borne spread to lungs, bones and liver. Moreover BBC are mostly lobular carcinomas but we report a rare case of Infiltrating Ductal Carcinoma (IDC) as the primary carcinoma and Lobular Carcinoma as the secondary.

**Case description:**

56 year old female who presented with a Stage IIIB fungating growth around 10x8cm in the lower inner, lower outer and upper outer quadrant of right breast since 6 months and a StageIIA 4x4cm tumour felt in the left breast in the upper inner and lower outer quadrant. Wedge biopsy of the right breast fungating mass showed Ductal Carcinoma and FNAC of the left breast lump suggested Lobular carcinoma which was confirmed on Histopathology after surgery.

Patient was subjected to Hormonal therapy (Tab Tamoxifen), chemotherapy (Cyclophosphamide, 5 FU, and Doxorubicin) followed by Radiotherapy.

Patient recovered well and shows no recurrence or signs of metastasis in the 8 months of follow up.

**Discussion and evaluation:**

Different histological subtypes with different grades of tumour in both breasts suggested two different synchronous primary tumours. Early detection of the contralateral tumour is of utmost importance emphasising the significance of breast self-examination. Screening tools like MRI have a greater sensitivity compared to Mammography. There are no clear treatment guidelines for bilateral breast cancer. Patients are often treated with bilateral mastectomy, with breast conservative surgery having unclear importance.

**Conclusion:**

Meticulous diagnosis and appropriate management help to improve the longitivity with an improved quality of life.

## Introduction

Bilateral Breast Carcinoma (BBC) is an uncommon presentation with an incidence of 2-5% of all breast malignancies (Chandrika et al. 2012). Understanding the various factors contributing the development of contralateral tumour is important to ameliorate its altered clinical course, exaggerated treatment course and cost, aggravated prognosis as compared to unilateral tumour.

Here is a case of a 56 year old female with bilateral breast carcinoma with different histology and different grade on either side, who was managed adequately and recovered well.

## Case report

A 56 year old female presented with a fungating growth in the right breast since 6 months which began as a small lump which gradually increased in size and ulcerated over time. There is no family history of breast cancer. On local examination, a 10X8cm fungating growth in the lower inner, lower outer and upper outer quadrant of right breast, fixed to underlying pectoral muscles with two, mobile, firm right anterior and central group of axillary lymph nodes and multiple satellite skin nodules over the chest.

**Figure 1:**
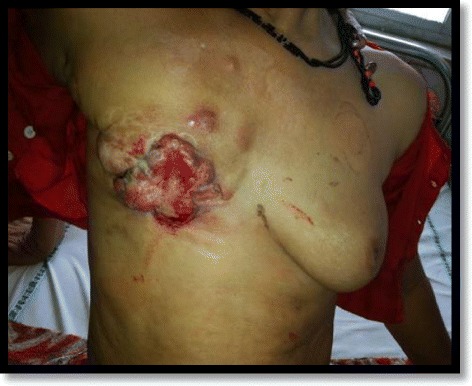
Clinical picture of the patient.

Another 4x4cm firm, mobile lump, felt in the left breast in the upper inner, central and lower outer quadrant, with no palpable axillary lymph nodes on the left side (Figure 1).

**Figure 2:**
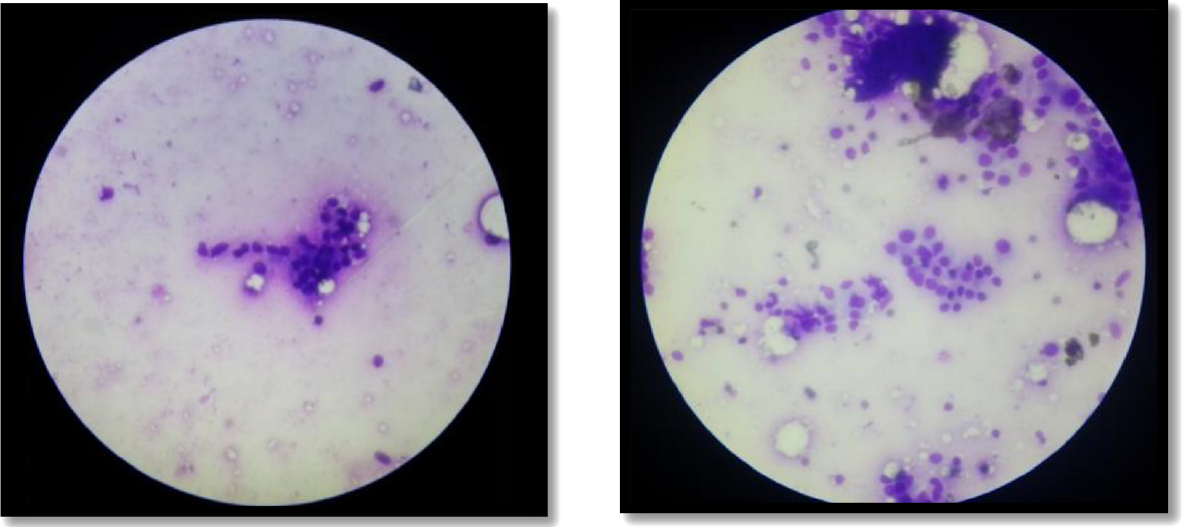
FNAC of left breast showing Lobular Carcinoma.

General physical examination and systemic examination were normal. Wedge biopsy of the right breast fungating mass suggested Infiltrating Ductal Carcinoma and FNAC of the left breast lump suggested Lobular Carcinoma (Figure 2).

Distant Metastasis to other organs was ruled out in view of a normal clinical examination, normal Ultrasound abdomen and normal chest X-ray.

**Figure 3:**
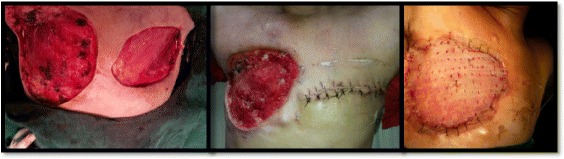
Intra-operative pictures of right toilet mastectomy with complete axillary dissection and left simple mastectomy.

Patient underwent Toilet Mastectomy for right breast with right axillary dissection and Simple Mastectomy for left breast in a single sitting, followed by Split Skin Grafting for raw wound over right breast region after 15 days (Figure 3).

**Figure 4:**
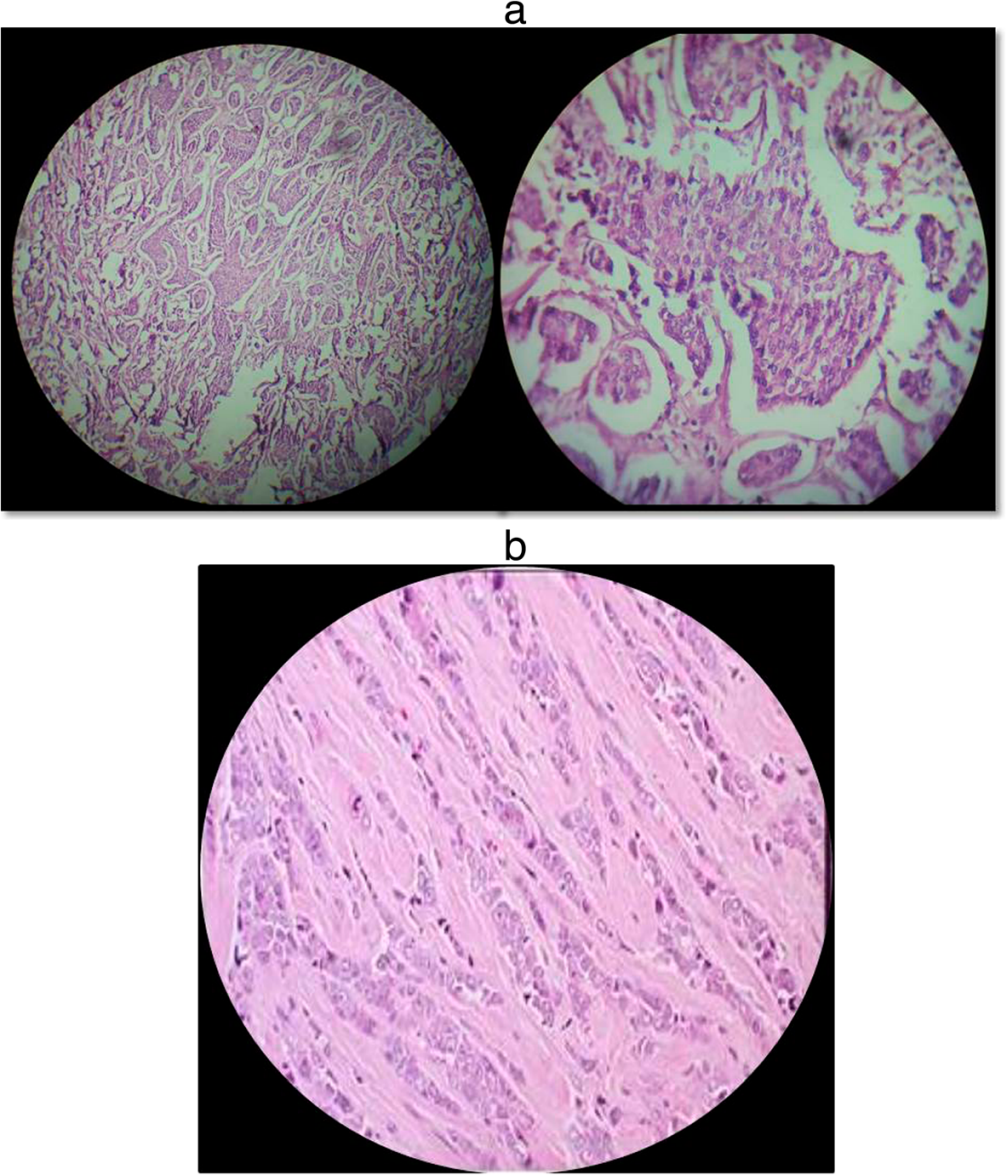
Histopathology. **a)** Right breast specimen showing Infiltrating Ductal Carcinoma. **b)** Left breast Specimen showing Lobular carcinoma.

Histopathology of the right breast specimen proved to be Infiltrating Ductal Carcinoma whereas of the left breast specimen proved to be Lobular carcinoma (Figure 4).

Specimens were tested positive for Estrogen and Progesterone Receptors and but negative for Her2neu receptor.

Final diagnosis of right sided Stage IIIB Infiltrating Ductal Carcinoma and left sided Stage IIA Lobular Carcinoma was made.

Patient received 6 cycles of chemotherapy with Cyclophosphamide, 5FU and Doxorubicin, and is currently on adjuvant hormonal therapy with Tamoxifen with external beam Radiotherapy to both sides.

**Figure 5:**
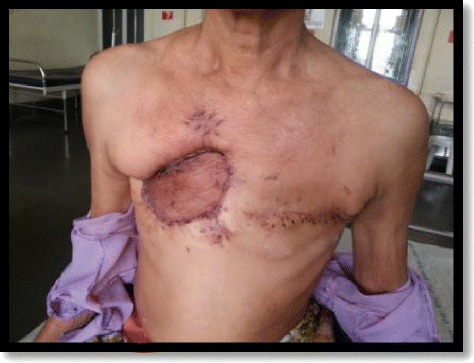
Patient in post-operative follow up.

Postoperatively patient has recovered well (Figure 5). On follow-up, clinical examination and PET scan shows no signs of recurrence after 8 months.

## Discussion

Bilateral Breast Carcinoma (BBC) is an uncommon presentation with an incidence of 2-5% of all breast malignancies (Chandrika et al. 2012). The second tumour in the contralateral breast can be either synchronous (within 6 months of the primary tumour) or metachronous (after 6 months of the primary) (Chandrika et al. 2012).

In our case the second lump in the left breast developed 3 months after the right breast lump and was completely ignored by the patient.

The exact etiology is not clearly defined (Chen et al. 1999) but among the various hypothesis suggesting risk factors for bilaterality of breast carcinomas; lobular carcinoma as the primary carcinoma is considered as an important factor. Women previously diagnosed with breast cancer, are at increased risk of developing contralateral breast tumour with a two to six times greater relative risk than developing a first breast cancer in general population (Kheirelseid et al. 2011; Chaudary et al. 1984). Other factors include a positive family history of breast cancer, genetic predisposition, a younger age at the diagnosis of the first primary breast cancer, inadequate treatment received for the first tumour and nulliparity (Chen et al. 1999; Kheirelseid et al. 2011).

A tumor in contralateral breast may represent either a second primary tumor or metastasis from first tumor. Various studies (Chandrika et al. 2012; Tuttle and Douglas 2004; Leis 1978) proposed guidelines, including Chaudary et al. (1984) who proposed a criteria to differentiate between a separate second primary and metastasis to the other breast; comprising of- demonstration of In Situ Disease on either side, both carcinomas with different histological types and different grades of cancer with no evidence of local, regional or distant metastasis. Generally in the absence of widespread systemic metastases, there is more likelihood of contralateral breast tumors being separate primary tumors.

In our case different histological subtypes with different grades of tumour suggested two different synchronous primary tumours.

Early detection of the contralateral tumour is of utmost importance emphasising the significance of breast self-examination. Screening tools like MRI have a greater sensitivity compared to Mammography (Kheirelseid et al. 2011).

Unlike unilateral breast cancer, there are no clear treatment guidelines for bilateral breast cancer. Patients are often treated with bilateral mastectomy, with breast conservative surgery having unclear importance. (Kheirelseid et al. 2011) In view of preventing bilaterality of tumours and with various recent breast reconstruction options, there has been dramatic increase in preference for prophylactic contralateral mastectomy for unilateral tumors (Kheirelseid et al. 2011).

Our management plan was based upon the grade of the individual tumours. The right breast had a fungating Stage IIIB tumour with involvement of the pectoralis major muscle. Therefore toilet mastectomy with excision of some fibres of pectoralis major muscle was done with a complete ipsilateral axillary dissection. Whereas a simple mastectomy in view of a stage IIA tumour on the left side sufficed.

There is no clear relationship between ER and PR positivity and Bilaterality of the tumour. But bilaterality is more commonly seen in cases with Her-2/neu overexpression (Kheirelseid et al. 2011).

Studies suggest that there was no significant difference in survival for patients with bilateral compared to unilateral tumour (Branica et al. 2010), but synchronous tumours was associated with poorer survival in comparison to metachronous tumours (Kheirelseid et al. 2011).

## Conclusion

In a case of bilateral synchronous breast carcinoma with different grades and histology; meticulous diagnosis and appropriate management helped to improve the longitivity with an improved quality of life.

## Consent

Written informed consent was obtained from the patient for the publication of this report and any accompanying images.
